# Automated annotation of chemical names in the literature with tunable accuracy

**DOI:** 10.1186/1758-2946-3-52

**Published:** 2011-11-22

**Authors:** Jun D Zhang, Lewis Y Geer, Evan E Bolton, Stephen H Bryant

**Affiliations:** 1 National Center for Biotechnology Information, National Library of Medicine, National Institutes of Health, Department of Health and Human Services, 8600 Rockville Pike, Bethesda, MD 20894, USA

## Abstract

**Background:**

A significant portion of the biomedical and chemical literature refers to small molecules. The accurate identification and annotation of compound name that are relevant to the topic of the given literature can establish links between scientific publications and various chemical and life science databases. Manual annotation is the preferred method for these works because well-trained indexers can understand the paper topics as well as recognize key terms. However, considering the hundreds of thousands of new papers published annually, an automatic annotation system with high precision and relevance can be a useful complement to manual annotation.

**Results:**

An automated chemical name annotation system, MeSH Automated Annotations (MAA), was developed to annotate small molecule names in scientific abstracts with tunable accuracy. This system aims to reproduce the MeSH term annotations on biomedical and chemical literature that would be created by indexers. When comparing automated free text matching to those indexed manually of 26 thousand MEDLINE abstracts, more than 40% of the annotations were false-positive (FP) cases. To reduce the FP rate, MAA incorporated several filters to remove "incorrect" annotations caused by nonspecific, partial, and low relevance chemical names. In part, relevance was measured by the position of the chemical name in the text. Tunable accuracy was obtained by adding or restricting the sections of the text scanned for chemical names. The best precision obtained was 96% with a 28% recall rate. The best performance of MAA, as measured with the F statistic was 66%, which favorably compares to other chemical name annotation systems.

**Conclusions:**

Accurate chemical name annotation can help researchers not only identify important chemical names in abstracts, but also match unindexed and unstructured abstracts to chemical records. The current work is tested against MEDLINE, but the algorithm is not specific to this corpus and it is possible that the algorithm can be applied to papers from chemical physics, material, polymer and environmental science, as well as patents, biological assay descriptions and other textual data.

## Background

Significant portions of the biomedical literature refer to chemical structures. For example, metabolites and small signaling molecules are crucial to life and well-studied, while many natural and synthetic products are examined in the context of drug discovery. The accurate identification and annotation of chemical names that are topically relevant to literature is a critical first step to establish links between scientific publications and the databases containing information about the chemical structure the name represents (*e.g*., molecular structures, measured biological activities, and drug information). Currently, manual identification is the preferred method for these chemical annotations as well-trained indexers can semantically understand and rank paper topics as well as recognize key terms; however, when considering the hundreds of thousands of new scientific articles published annually, an automatic annotation algorithm with high precision and relevance is a useful adjunct to manual annotation.

Current studies [1-13] on the text mining of small molecule names focus on the named entity recognition (NER) of chemical descriptors, including systematic chemical names such as IUPAC names and common names. Dictionary and rules (DR) based methods and statistical machine learning (ML) methods are two major approaches in this area. In 1992, Chowdhury and Lynch [1,2] developed a dictionary and rule based semiautomatic method to convert chemical texts into structure representation by morphological analysis and dictionary lookup. In 1999, Wilbur, *et. al*. [3] compared three NER methods (one rule and dictionary based method and two Naïve Bayes statistical methods) to recognize chemical terms in biological text, and concluded that an integrated method might perform best. Hettne and co-workers [4,5] generated dictionaries identifying small molecules and drugs in text, and found that a dictionary generated from a reliable single source, ChemIDplus [14] performs as well as a dictionary from combined multiple sources. Wren [6] evaluated a first order Markov Model for its ability to distinguish chemical names from words. Klinger [7] implemented a new machine learning approach based on Conditional Random Fields (CRF) to detect IUPAC and IUPAC-like chemical names in the scientific literature and obtained good performance: an F measure of 85.6% on a MEDLINE [15] corpus. Corbett, Jessop and co-workers [8-11] performed studies on chemical name mining on text and developed OSCAR4 [11], an open source system to identify chemical names in scientific articles. Kolarik and her co-workers [12,13] analyzed chemical terminology resources and generated an annotated text corpus for evaluation of dictionaries. Recently, Zhou and his co-workers [16] designed and implemented a chemistry text hybrid search engine to combine both chemistry text and structure searching in literature. Generally speaking, the ML based methods perform extremely well in recognition on IUPAC or IUPAC-like chemical names, but not as well on common names. On the other hand, the DR based methods can identify both IUPAC and trivial names, but it is not possible to identify names not in the dictionary. Nevertheless, both approaches concentrate on the identification of chemical names but focus less on ranking the annotations for relevance, which is a key goal of this study. For example, a chemical mentioned in a metabolic pathway paper may not be the molecule that can trigger the pathway, rather it might be an inactive chemical compound, related to the methodology, or a substrate mentioned in a longer protein name or gene name. Banville [17] addressed a similar problem: how do you find documents of relevance to a chemical instead of simply finding the chemical name present in a document?

Medical Subject Headings [18] (MeSH) annotation, which is performed by trained curators of the National Library of Medicine (NLM) to index and categorize articles in the MEDLINE databases, is a reliable source for users of MEDLINE and PubMed [19] to obtain relevant and accurate scientific term annotations. To aid human indexing of the MEDLINE database, NLM developed an automatic indexing system [20-22], the Indexing Initiative system (IIS), to identify candidate MeSH concepts in papers being indexed, helping to speed the manual annotation of the biomedical literature.

In recent years as the volume of literature has grown, the accuracy and relevance of retrieved information have become key performance indicators of on-line chemical databases. A single query can retrieve many thousands of records, making it essential that the top ranked results are highly relevant to the user. Additionally, it is very useful to link records from one database to those in another. In the NCBI Entrez query system [23], MeSH plays a vital role in both improving query performance and for making links between databases. For example, the MeSH vocabulary allows for synonym expansion in PubMed queries, precise querying of chemical names, and allows the linking of abstracts to the small molecule records in the PubChem [24] database; however, manual annotation of MeSH onto PubMed abstracts can have a time lag of a few months and other sources of scientific literature may not have MeSH annotation at all. In these situations, it may be useful to have an algorithm for automatic annotation of MeSH terms.

In this article, we present an implementation of an automated chemical name annotation system based on the MeSH controlled vocabulary called MeSH Automated Annotations (MAA). The primary aim of MAA is to reproduce the MeSH term annotations created by curators.

## Methods

### 1. Corpus generation

The annotated text corpus is generated directly from MEDLINE, with PubMed identifier (PMID) ranging from 16200042 to 17342794. In order to increase the recall of automated annotation, we only select entries with both title and full abstract available, giving a total of 261,227 MEDLINE abstracts inside the corpus. Each paper in the corpus has been annotated by the NLM indexers. These human annotations of chemical names are used as the "gold standard" for comparison with the various versions of MAA described in this paper. We performed spot checking of randomly selected manual annotations and found they are reliable to be used as standards. However, the NLM indexers' aim is to annotate topic-related chemical entities, thus the selections depend on the indexers' understanding of the topic of a paper. It is nontrivial to tell if the unselected chemical entities are valid or not. Nevertheless, an automated annotation system should provide improvements on the possible errors of manual indexing. A randomly selected data set, which contains 26,123 abstracts, was selected to test our MAA program. The remaining abstracts were used as a training set to obtain statistics used to set thresholds for various filters used in the algorithm.

### 2. MeSH chemical dictionary generation

MeSH is a controlled vocabulary thesaurus from the NLM used to help index the biomedical literature. MeSH is organized in a hierarchical tree where each scientific concept is either a node or leaf of the tree. Scientific concepts include a MeSH heading (being the most common name used to refer to the concept), synonyms, and inflectional MeSH term variants. The parts of the MeSH tree associated with chemicals is composed of two parts, the 'Chemicals and Drugs' branch of the MeSH hierarchy and an independent set of supplementary concept records (denoted as MeSH substances). Each MeSH substance is mapped to at least one MeSH term. Chemical compounds of relatively recent biomedical interest are either appended to the MeSH tree or added to the MeSH substances. The MeSH chemical vocabularies are used as the basis of our dictionary. In the following text, we will use the phrase 'MeSH term' to refer to any MeSH heading, MeSH term, or MeSH substance under or mapped to the Chemicals and Drugs branch of MeSH.

### 3. Statistical terminology for evaluation of MAA

The objective of our MAA system is to find relevant MeSH terms in abstracts. As mentioned previously, in an abstract there may be many MeSH terms found in the text, but not all of these are related to the topic of the document. The human MeSH indexer annotates these relevant MeSH terms by reading and understanding the subject material. Thus, we compare our MAA system to the manual annotations of the MeSH indexers. In our approach, we intend to reproduce the MeSH indexers' annotation by extracting relevant terms and filtering out unimportant MeSH terms. Using this manual indexing as the standard, the terminologies used for evaluation of MAA are:

True positive (TP) match -- A MeSH term found by both MAA and manual indexing.

True negative (TN) match -- A MeSH term not found by either MAA or manual indexing.

False positive (FP) match -- A MeSH term found by MAA but not by manual indexing.

False negative (FN) match -- A MeSH term found by manual indexing but not by MAA.

Note that the false negatives include terms that are not in the title or abstract of the documents as the MeSH indexers have access to the complete document. These terms cannot be found by MAA as the algorithm does not have access to the complete document. In the following figures and discussions, the total FN matches were separated into two groups: the group "In Text, Not Found" refers to MeSH annotations where the MeSH terms are in the abstract but are not found by MAA and "Not in Text" to refer to instances where the MeSH term is not present in the text. In the latter case, terms are typically found in the body of the paper.

Precision and recall are calculated as following:

$$Precision=\frac{TP}{TP+FP}$$

$$Recall=\frac{TP}{TP+FN}$$

$$F\;Measure=\frac{2\times Precision\times Recall}{Precision+Recall}$$

### 4. Chemical Tokens and Rules

A Chemical token is a string used to build chemical names. In this study, a chemical token dictionary was created to generate chemical morphemes. These chemical tokens are made by dissecting chemical names at white space and other separators. The chemical names are taken from MeSH terms and PubChem Compound synonyms, encompassing over 31 million chemical records. We chose the PubChem as a source of chemical names as it is a large database of small molecule structures (> 30 million), including depositions from many popular chemical databases, such as ChemIDPlus, ChEBI, ZINC, etc. MeSH was selected as it is a comprehensive controlled vocabulary that has been applied extensively in biomedical literature indexing, including the indexing of most PubMed records, making it likely to contain a significant subset of biomedically interesting chemical names. However, the MAA algorithm is not limited to these sources - in particular, a more detailed controlled vocabulary may improve the results of the algorithm. After the tokens are generated, two English novels "*Jane Eyre*" and "*Pride and Prejudice*" are used to filter out common English words from the tokens. Numbers, numerical identifiers, single characters and special characters are removed. Overall, there are total 326,610 chemical tokens stored in our token dictionary. These chemical tokens, along with name decision rules, were used to check if a MeSH term embedded in text is a full name or a sub-string of another name. In MAA, a MeSH term and two tokens before and after the MeSH term are analyzed. If the combination of the MeSH term and the tokens fulfill one of the name decision rules, the MeSH term will be marked as a likely substring of a complete chemical name.

## Results and Discussion

There are several steps in the MAA algorithm. The first step is free-text matching of the MeSH vocabulary to the MEDLINE abstracts. To measure the performance of this step and subsequent steps, we compare the results to manual annotations of these abstracts done by the MeSH indexers. In this comparison, we take into account that in some cases the indexer used a more general term (aka a "relative node") than the precise name of the chemical, such as "Benzodiazepines" instead of "Diazepam." Note that it is not possible for the algorithm to find all terms annotated by the indexers as the indexers have access to the complete paper and the algorithm does not.

Subsequent steps in the algorithm attempt to reduce the number of false positives, which are the matches found by the algorithm but not indexers. The first step, the "MeSH Filter" eliminates MeSH records that do not have an associated chemical structure. The second step, "Tokens and Rules", discards partial matches to terms that follow chemical nomenclature rules or have additional chemical name tokens. The third step, "Protein and Gene Names", screens out protein and gene names as these names can contain the names of chemicals. Finally, the "TP filter" eliminates matches using MeSH terms that are also common English terms, such as "lead."

**Figure 1:**
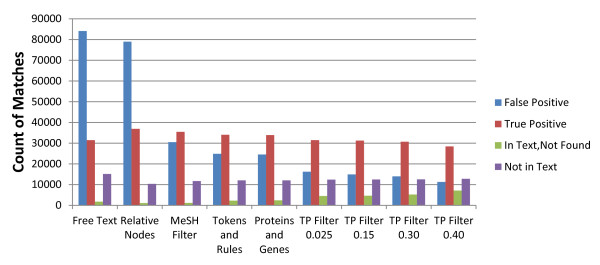
**Comparison of MeSH automated (MAA) and manual indexing annotations with a series of algorithmic filters added**. From left to right, filters are applied cumulatively. "False Positives" are matches found by the algorithm but not in manual indexing. "True Positives" are found by the algorithm and in manual indexing. "In text, not found" are found in manual indexing, but not by the algorithm. "Not in text" are manually indexed terms not found in the abstract.

The comparison between the various steps in MAA and manual indexing is depicted in Figure 1 and will be discussed in detail below. From left to right, each group of bars indicates the matching results for different steps in the algorithm. Within each group there are 4 bars, starting from the left:

1. False positive (blue): MeSH terms found in an abstract by MAA but not found by manual indexing.

2. True positive (red): MeSH terms found in an abstract by MAA and also found by manual indexing.

3. In Text, Not found (green): MeSH terms present in the abstract and found by manual indexing but not found by MAA.

4. Not in text (purple): MeSH terms not present in the abstract but found by manual indexing. As mentioned earlier, some terms are found in the body of a paper and not in the abstract. Since the MAA algorithm does not have access to the body of the paper, it is unable to find these terms. The value shown in the figure is likely an upper bound as it is possible that the algorithm may not find a term due to various potential issues (e.g. punctuation, spelling, unknown synonyms, etc.).

### 1. Free-Text MeSH matching

**Figure 2:**
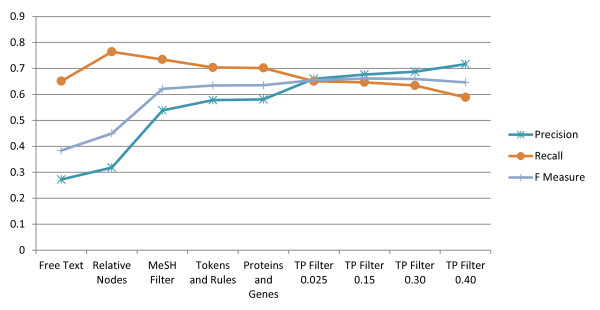
**The recall, precision and F measure of MeSH automated annotations (MAA) on the titles and abstracts of the test corpus with a series of algorithmic filters added cumulatively**.

As displayed on Figure 1, the free-text MeSH string matching, if used to annotate small molecules directly, generates a significant number of false-positive cases compared to the MeSH indexers' annotations. More than 70% of the MeSH terms appearing in the text were not annotated by indexers (see Table 1 for values and Figure 2 for graphs of the precision, recall and F measure). By inspection, it appears that in most cases the MeSH term may either be a text fragment of another scientific concept (e.g., many protein names include aspects of a chemical name) or the MeSH term is simply present but not relevant to the paper context (e.g., used in a descriptive or comparative sense, as a reagent in an experiment, etc.). Thus, in order to annotate a chemical term accurately, algorithmic filters need to be applied to the free-text matches. In some cases, the indexer missed annotating a valid chemical name. However, this category was not examined as it would have required another standard of truth, which was unavailable for the large set of abstracts considered in this study.

**Table 1:** The values of precision (P), recall (R) and F value (F) of MeSH Automated Annotation (MAA) on title and full abstract; title, first and last sentences of abstract and title-only annotations respectively, with a series of algorithmic filters added cumulatively.

	MAA on title and full abstract			MAA on title, first and last sentences of abstract			MAA on title-only		
	
	P	R	F	P	R	F	P	R	F
Free Text	0.272	0.651	0.384	0.401	0.399	0.400	0.545	0.297	0.385
Relative Nodes	0.318	0.764	0.450	0.483	0.511	0.497	0.627	0.385	0.477
MeSH Filter	0.538	0.735	0.621	0.710	0.480	0.573	0.861	0.366	0.513
Tokens&Rules	0.578	0.704	0.635	0.750	0.451	0.563	0.907	0.331	0.485
Proteins&Genes	0.581	0.702	0.635	0.759	0.443	0.560	0.914	0.328	0.482
TP Filter 0.025	0.660	0.651	0.655	0.828	0.409	0.547	0.942	0.304	0.460
TP Filter 0.15	0.677	0.647	0.661	0.844	0.405	0.548	0.949	0.302	0.458
TP Filter 0.30	0.687	0.634	0.660	0.852	0.399	0.543	0.952	0.297	0.452
TP Filter 0.40	0.716	0.589	0.646	0.871	0.371	0.521	0.960	0.277	0.430

### 2. Using relative nodes in the comparison

In comparing results between the MAA and the manual indexers, tree-node expansion is applied to the results from the MAA system. The term "tree-node" comes from the hierarchical tree structure of the MeSH thesaurus. Examination of the MeSH annotations in MEDLINE finds that the indexers will sometimes select a higher level node in the MeSH tree than the nodes that correspond exactly to the chemicals mentioned in an abstract. For example, they may select a more generic term "Penicillins" instead of the "Penicillin G" and "Penicillin V" mentioned in a paper. This use of higher level ("super-concept") nodes also happens for MeSH substances as they are manually mapped to nodes in the MeSH tree. Therefore, for a given MeSH substance or MeSH term, we include its assigned MeSH tree node and/or super-concept node, respectively. This tree-node expansion significantly increases the number of matches between manual indexing and our MAA algorithm (Figure 1), while also increasing the recall and precision due to the increase in the number of true positives (Table 1 and Figure 2).

### 3. Improving free-text MAA by adding filters

#### 3.1. MeSH filter: using terms with associated chemical structure

From the entire set of chemical MeSH terms, MeSH terms representing small molecules are extracted to generate a chemical dictionary. This constraint is implemented by selecting MeSH terms which can map to PubChem (http://pubchem.ncbi.nlm.nih.gov/) CIDs, which identify unique small molecule structures. This filter removes almost 65% of the total MeSH terms (see Table 2, the numbers of MeSH terms change from 521 k to 178 k). Filtered terms include protein names, category names, non-specific names and chemical names which cannot be represented by molecule structures. After filtering out terms, we expand the MeSH dictionary by adding 541 chemical formulas of inorganic compounds as synonyms of associated MeSH concepts. All of these chemical formulas are from Wikipedia [25] and were manually verified. This addition increases the ability of MAA to recognize chemicals such as 'KOH' and 'NaCl' if no compound common name is mentioned in the text. After updating our dictionary, we performed another free-text match and compared the results with manual indexing. The results are shown in the second group of bars labeled "MeSH Filter" in Figure 1. Compared to "Relative Nodes", the number of FP cases after adding the MeSH Filter drops from 79 K to 30 K, and the number of TP cases decreases less than 1 K. The performance of MeSH filter, indicated by precision, recall and F measure is displayed in Table 1. The precision jumps from 0.32 to 0.54, and loses 0.03 recall. As a result, the F measure has an increase from 0.45 to 0.62. The MeSH filter assures that all extracted MeSH terms by MAA are entries in PubChem. It thus implicitly links chemical names in literature to variant features of the PubChem database, such as chemical structures, properties and bioactivities etc.

**Table 2:** The number of MeSH terms in the dictionary after each filter is applied.

		Numbers of MeSH Term
Total MeSH Terms		521,663

MeSH Terms with PubChem CID mapping		178,110

Add Inorganic Formulas		178,651

Add TP ratio filter	0.025	178,250
	
	0.15	177,796
	
	0.3	174,580
	
	0.4	174,385

#### 3.2. Tokens and Rules: removing false positive annotations by syntactic analysis

MeSH terms that are sub-strings of another entity name is one reason for false-positive annotation. The chemical tokens and chemical name decision rules (introduced in Methods part 4) were used to decide if matched MeSH terms are full names or substrings.

Some of the applied rules are listed below:

[1]. If two words in front of a matched MeSH term are both chemical tokens, the MeSH term is treated as a FP annotation. This rule by itself yields a 0.25% increase in precision, a 0.34% decrease in recall and a 0.04% increase in F measure;

[2]. If one word in front of a matched MeSH term is a chemical token, the MeSH term is treated as a FP annotation. This rule by itself yields a 1.3% increase in precision, a 1.6% decrease in recall and a 0.24% increase in F measure;

[3]. If one word behind a matched MeSH term is a chemical token, the MeSH term is treated as a FP annotation. This rule by itself yields a 2.2% increase in precision, a 1.9% decrease in recall and a 0.68% increase in F measure. Note that the F measure is the harmonic average of precision and recall, which is why the change in F measure is not exactly the difference between the change in precision and recall.

Using more than two tokens before and after the MeSH term did not yield any improvements. For example, if the algorithm checks 3 tokens before and after the matching MeSH term, the recall decreases 0.72% and precision decreases 0.87%.

In addition to these name decision rules, we also created several prefix and suffix rules to check whether a matched term is FP annotation. For example, if the token '*poly*' is the prefix of a MeSH term, this MeSH term is treated as a FP annotation, yielding a 0.12% increase in precision, 0.03% decrease in recall and 0.07% increase in F measure; if '*ase*' is the suffix of a MeSH term (except '*release*' and '*base*') the MeSH term is treated as a FP annotation, yielding a 1.2% increase in precision, 0.15% decrease in recall and 0.75% increase in F measure. These rules were primarily heuristic in nature and were developed by manual examination of the annotations.

It is possible to apply hundreds of rules to increase the precision of MAA. However the recall decreases as each rule applied. It is nontrivial to decide which rules should be used. In the MAA system, we select rules according to the computed F measure. If we obtained a relatively significant positive increment of F measure by applying a rule, the rule was kept.

The following is an actual annotation of a PubMed abstract (PMID 16704345) to show how this filter works:

*...Related enzymes are the ATP-dependent **benzoyl-CoA **reductase and the ATP-independent **4-hydroxybenzoyl-CoA **reductase. Ketyl radical anions may also be generated by one-electron oxidation as shown by the flavin **adenine **dinucleotide (FAD)- and [4Fe-4S]-containing **4-hydroxybutyryl-CoA **dehydratase...*.

The bold words are mapped MeSH terms, and the words underlined are chemical tokens found before or after MeSH terms. For example, according to the rules, "*flavin-**adenine**-dinucleotide*" is the complete name and MeSH term "adenine" is just part of this name. Thus, the MeSH term "adenine" is regarded as a false-positive by our MAA program.

In Figure 1, the fourth group of bars indicates the change after adding the "Token and Rules" filter. Compared to previous group of bars (MeSH Filter), the blue bar (false-positive annotation) dropped more than 5000 and red bar (true-positive) only lost 1300 annotations. Please see additional file 1 for a detailed description of chemical token generation and chemical name decision rules.

#### 3.3. Protein and gene names: removing MeSH terms that are sub-strings of protein, gene and non-chemical MeSH terms

Chemical terms are a common part of protein names, such as "benzoyl-CoA reductase" and "4-hydroxybutyryl-CoA dehydratase" shown above. When these protein names are mentioned in text, it is likely that the topic of the paper is the protein instead of the prefix chemicals. To address this issue, we created a group of "negative vocabularies" to collect names that contain MeSH terms as sub-strings. In the MAA algorithm, if a term in the negative vocabularies is found in text, then its sub-string will not be annotated if this sub-string is a MeSH term. The protein and gene names are collected from MeSH and the NCBI Entrez Gene database. The performance of this method depends on the completeness of the negative vocabularies. It is not possible to construct a complete dictionary, as new names are generated every day. In Table 1, we can see that this filter results in only a small increase of the F measure at best. This is because the "token and rules" filter and the "protein and gene" filter are not mutually exclusive: some rules in section 3.2 already remove many protein names. If "tokens and rule" and "protein and gene name" filters are applied independently on the same corpus, the former will yield 2.6% more precision and 0.5% more F measure. This result is possibly due to the fact that the "token and rule" filter attempts to be a superset of the "protein and gene name" filter. Nevertheless, the protein and gene name rule is still useful in removing false positive matches for certain protein names.

#### 3.4. TP filter: removing MeSH terms with low TP ratios

Some MeSH terms, such as the dental sealant "Conclude" (also known as "Concise"), have a high false positive rate due to nonspecific matching. These terms are filtered out to improve match statistics. To do this, we pre-calculated the true positive ratios of each MeSH term using free-text string matching on the training set. A binary value (1 or 0) was assigned to each MeSH term to indicate if it exists or not in the MEDLINE abstract. If a term was mentioned multiple times in an abstract, it was still counted as 1. The ratio of TP annotation for a specific MeSH term was calculated by the number of times the term was applied during manual indexing divided by the count of abstracts with free text matches. This ratio is used to measure the propensity of a MeSH term to be correctly annotated in text. Some MeSH terms with their TP ratios are listed in Table 3. Common chemicals such as 'water' and 'glucose' tend to have a less than 50% TP ratio. The term 'lead' has only 11% TP ratio, which indicates in only 11 out 100 papers, 'lead' is indexed as a chemical element. Additional term types with low TP ratios include homonyms of common English words, such as 'link' and 'monitor', or acronyms such as 'CI-2' that have only a few characters. Using the TP ratio, one may set up a tunable threshold to eliminate non-specific MeSH terms in automatic annotation.

**Table 3:** Selected MeSH terms with TP ratios ranked from lowest to highest based on a 230 K abstract corpus (Total 260 K abstracts minus 26 K testing corpus).

MeSH Term	Number of Abstracts Appeared (A)	Number of Abstracts Annotated by Curator (C)	True-Positive Ratio = C/A
link	2024	0	0

monitor	1889	0	0

at 10	1535	0	0

counter	585	0	0

CI-2	201	0	0

conclude	5677	1	0.00018

advantage	1723	1	0.00058

lead	6229	774	0.110

water	11155	4898	0.305

glucose	7282	3145	0.302

dexmedetomidine	95	2	0.979

ivabradine	32	32	1

Once a threshold ratio is selected, MeSH terms with a ratio lower than the threshold will not be annotated on the testing data set. Selecting a reasonable threshold will remove false-positive annotations and increase the precision of MAA while not significantly reducing the recall. For example, if the threshold is set to 0.025, there are only 401 total MeSH terms eliminated, but nearly 8297 FP annotations are removed (in Figure 1, this difference is shown by the blue bar when going from 'Proteins and Genes name' to 'TP ratio 0.0025'), while 2466 TP annotations are lost (In Figure 1, this difference is shown by the red bar when going from 'Proteins and Genes name' to 'TP ratio 0.025'). In our study, the thresholds are adjusted from 0.025 to 0.4 to show the trade off in recall as precision increases. Thresholds larger than 0.5 were not evaluated, since the MAA will lose more TP annotations than FP annotations. The best threshold ratio by F measure is between 0.1 and 0.2 (see Figure 2). This TP ratio filter provides a degree of tunable accuracy for the MAA system.

### 4. Term position in the text

**Figure 3:**
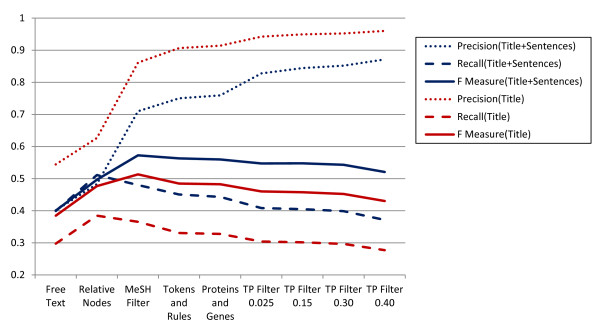
**The recall (dashed line), precision (dotted line) and F measure (solid line) of MeSH Automated Annotation (MAA) on titles only and on title and selected abstract sentences of the test corpus with a series of algorithmic filters added cumulatively**. Sentences = first and last sentences of abstract.

The title is often a summary of a paper. In the abstract, the author often mentions objectives in the first sentence (FT) and conclusions in the last sentence (LT). The appearance of a chemical name in these parts of an abstract is likely to indicate a high degree of relevance. We performed MAA on the title, FT and LT of the abstracts and then again just on the title of the abstract to see if we could obtain higher precision. The results are presented in Figure 3 and Table 1. In the title-only annotation, MAA could provide the 96% precision if the TP filter threshold is set to 0.4. At this filter level, there is greater than 27% recall on the corpus. Eliminating the TP filter for title-only annotation yields 91% precision with 33% recall. This may be because all words in the title are relatively important, reducing the necessity of the TP filter to remove non-specific MeSH terms. For an information retrieval task that requires a high degree of specificity, MAA on the title-only is a reasonable selection. Including the FT and LT in MAA yields less precision than title alone MAA, but with better precision than MAA on the entire abstract.

### 5. Comparison with other studies

In Table 4, the performance of MAA is compared to similar studies that matched MeSH to text. The top two rows in Table 4 list results from Hettne [4] and Kolarik [13]. As a gold standard, Hettne and Kolarik used a text corpus with Kolarik's manual annotations of chemical terms, which were not restricted to the MeSH vocabulary. We applied MAA to Kolarik's testing corpus (2009 version, containing 100 full abstracts). The dictionary used in MAA is a combination of MeSH tree chemical terms (MeSH C) and MeSH substances (MeSH S), but both Hettne and Kolarik separated these vocabularies. We first perform free-text matching of the MeSH dictionary to the Kolarik's corpus. The performance is very similar to Kolarik's, which were also performed using free-text matching. Then we applied each filter cumulatively as we did in our testing set in Section 3 of this paper. Once the TP filter threshold was set to 0.4, the performance we obtained is quite similar to those of Hettne's work, in which he used a "term disambiguation" pipeline to filter out some MeSH terms.

**Table 4:** The comparison of precision (P), recall (R), and F measure (F) of this work (MAA) with those of other studies.

	Corpus	Dictionaries	Filters	P	R	F
Hettne	Kolarik's Corpus	MeSH C MeSH S	Term Disambiguation	0.75	0.22	0.34
						
			Term disambiguation	0.83	0.07	0.13

Kolarik	Kolarik's Corpus	MeSH C MeSH S	Free Text	0.34	0.27	0.30
					
			Free Text	0.44	0.10	0.16

MAA (this work)	Kolarik's Corpus	MeSH C+S	Free Text	0.44	0.37	0.41
					
			MeSH Filter	0.61	0.34	0.43
					
			Token & Rule	0.66	0.33	0.43
					
			Protein & Gene	0.65	0.32	0.43
					
			TP Filter 0.025	0.73	0.28	0.41
					
			TP Filter 0.15	0.76	0.27	0.40
					
			TP Filter 0.30	0.79	0.26	0.39
					
			TP Filter 0.40	0.77	0.23	0.35

NLM's MTI*		All MeSH		0.29	0.55	0.38

MAA (this work)	A and T	MeSH C+S	TP Filter 0.15	0.68	0.65	0.66
			
	1S, LS and T		TP Filter 0.15	0.84	0.40	0.55
					
	T		TP Filter 0.15	0.95	0.30	0.46

The filters of MAA are applied cumulatively from "Free Text" to "TP Filter 0.4".

*See reference [20], this work use 273 articles with full text

MTI: Medical Text Indexer

MeSH C: MeSH tree chemical terms

MeSH S: MeSH substances

A: Abstract

T: Title

1S: First sentence of abstract

LS: Last sentence of abstract

MAA has a precision range from of 0.44 ~ 0.79 with different filters, which is better than Kolarik's precision range of 0.34~0.44 (for MeSH C and MeSH S, respectively). However, the MAA gives a higher recall range (0.23 ~ 0.37) than Hettne's (0.22~0.07) or Kolarik's (0.27~0.10). The best F measure which MAA generated is 0.43, which is better than the F-measure taken from either work (maximum of 0.34). Overall, the MAA results are closer to Kolarik's results if no filters applied and Hettne's results if TP threshold set to 0.4. However, as shown in Section 3.4, the higher TP threshold doesn't necessarily produce the better performance as ranked by F measure. When examining our 26123 abstracts testing set, the best performance of MAA was obtained when TP threshold was set to 0.15 and, when examining Kolaik's corpus, it was without applying the TP filter. This is consistent with results on our test corpus; while the TP filter significantly increases precision, it does so at the cost of recall.

The bottom rows of Table 4 show results from our MAA system and the Medical Text Indexer (MTI), which was developed for NLM's Indexing Initiative system (IIS) and whose goal was to provide suggested annotations to MeSH indexers. The results of MTI are not restricted to chemical names, so we cannot directly compare the results of MTI to MAA, but we include the results for reference. When MTI lists up to 25 recommendations for each article from a 273 articles corpus, it provided a recall of 0.55 and a precision of 0.29.

## Conclusion and Future Application

In this article, we present the design and implementation of an automated chemical name annotation system (MAA). This annotation system uses the MeSH controlled vocabulary applied to biomedical abstracts from MEDLINE. To avoid false positive annotations, we implemented filters to allow for tunable accuracy. The maximum precision obtained was 96% with 28% recall when performing MAA on titles of the abstracts. The best performance of MAA as measured with the F statistic was 66%, which required applying all filters (including the FP filter with a threshold of 0.15). The MAA system compared favorably to other chemical name retrieval studies. The current work is tested against MEDLINE, but the algorithm is not specific to this corpus and it is possible that the algorithm can be applied to papers from chemical physics, material, polymer and environmental science, as well as patents, bioassay descriptions and other textual data. Accurate MeSH annotation and text mining can help researchers not only to identify important chemical names in abstracts, but also match unindexed and unstructured texts to chemical records.

## Competing interests

The authors declare that they have no competing interests.

## Authors' contributions

JDZ carried out the study and wrote the manuscript. LYG helped conceive, design and coordinate the study. LYG also revised the manuscript. EEB helped conceive and design the study. EEB also revised the manuscript. SHB supervised and helped conceive and design the study. All authors read and approved the final manuscript.

## Supplementary Material

Additional file 1 **Details of token generation and rules**. Detailed description of chemical token generation and chemical name decision rules. Click here for file
