# Supplementary material for: Automated annotation of chemical names in the literature with tunable accuracy
Source: J Cheminform. 2011 Nov 22;3:52. doi: 10.1186/1758-2946-3-52 (PMC3281788; doi:10.1186/1758-2946-3-52)
Supplement: Additional file 1 — Details of token generation and rules. Detailed description of chemical token generation and chemical name decision rules. [file 1758-2946-3-52-S1.PDF]

Automated annotation of chemical names in the literature with tunable accuracy

Jun D. Zhang, Lewis Y. Geer*, Evan E. Bolton and Stephen H. Bryant

National Center for Biotechnology Information, National Library of Medicine, National Institutes of Health, Department of Health and Human Services, 8600 Rockville Pike, Bethesda, MD 20894, USA.

Supplementary Data

Supplementary

1. Chemical Token Generations

In this paper, a chemical token is defined as a string of English characters used to build chemical names. There should be no space or special characters in a token. The chemical tokens are generated by dissecting chemical names at white space and other separators. The chemical names are taken from MeSH terms and PubChem Compound synonyms, encompassing over 31 million chemical records. After the tokens are generated, two English novels "*Jane Eyre*" and "*Pride and Prejudice*" are used to filter out common English words. Numbers, numerical identifiers, single characters and special characters are removed. Overall, there are total 326,610 chemical tokens stored in our token dictionary. For example:

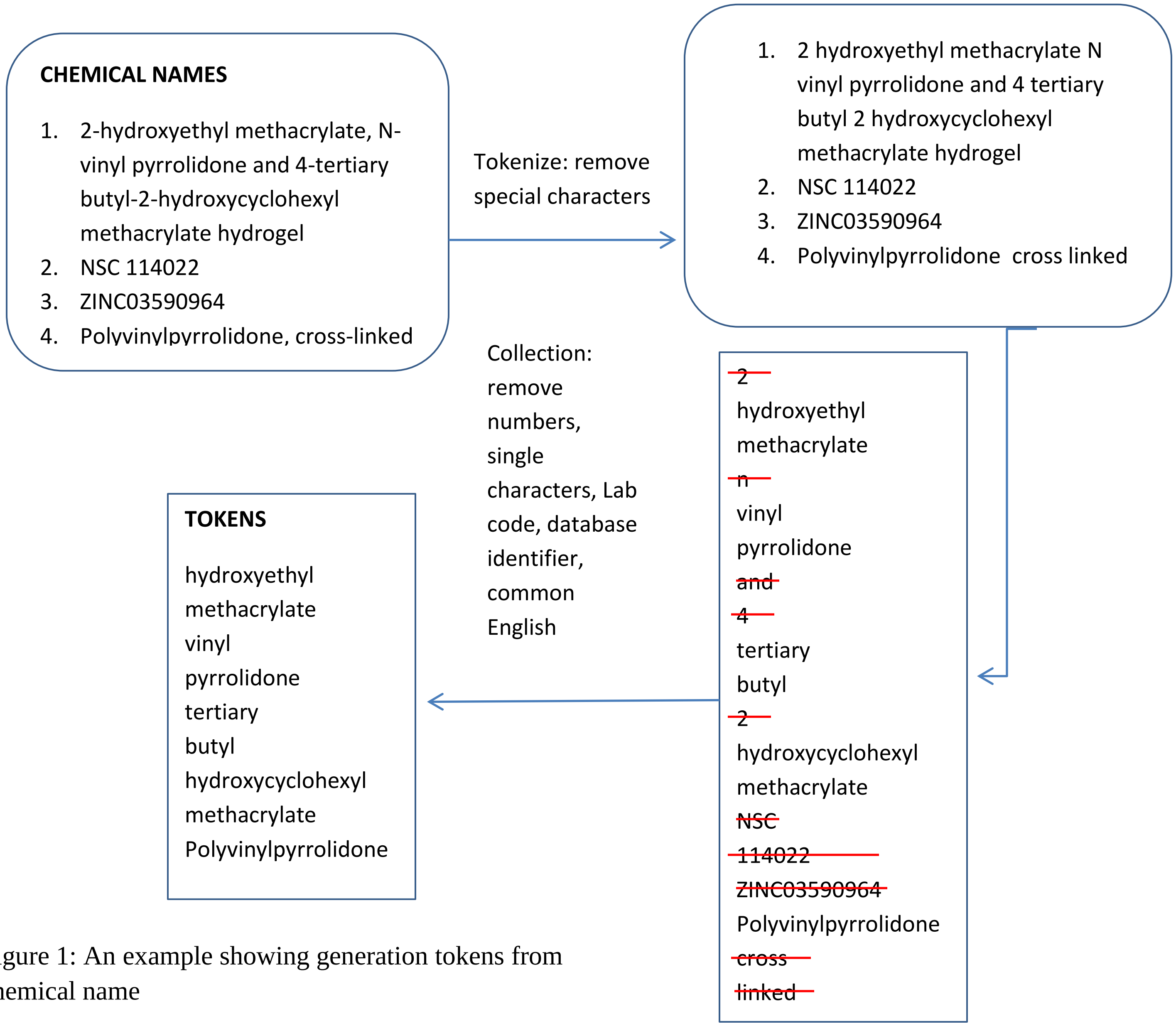

Figure 1: An example showing generation tokens from chemical name

2. Rules Applied

In MAA, a matched MeSH term and two tokens before and two tokens after this MeSH term are analyzed. If the combination of the MeSH term and the tokens fulfill one of patterns of name decision rule, the MeSH term will be marked as a likely substring of a complete chemical name. All rules are listed below.

Format: F2 F1 M E1 E2

M: Matched MeSH term

F1: The token in front of the matched MeSH term

F2: The token in front of the F1

E1: The token behind the matched MeSH term

E2: The token behind the E1

N: Numerical expression in chemical name

T: Chemical token

.

Table 1. The patterns of matched MeSH term with its front and behind 2 words

F2	F1	M	E1	E2
N	N	M	T	
T	T	M	T	T
T	N	M	N	T
N	T	M	T	N
	T	M		

In Table 1, there are 5 front patterns for F1 and F2, and 4 behind patterns for E1 and E2. Any of front or behind patterns can be found on text individually or combined. There are total (5+1)x(4+1)-1 = 29 combinations (remove M only combination).

3. Numerical expression in chemical name
   (1). Numbers (1-20).
   (2). Greek characters.
   (3). Hemi, semi, mono, uni, sesqui, di, bi, bis, tri, tert, tetra, quadri, penta, quinqui, hexa, sexi.
4. Additional rules

There are some independent rules defined:

(1). If E1 is end with character “ase” or “ases”, but E1 is not base, bases, release, and releases, the M is regarded as a substring.

(2). If F1 is start with character “poly”, the M is regarded as a substring.

(3). If E1 is end with character “analogy” or “analogies”, the M is regarded as a substring.

(4). If E1 is “calcium”, “sodium” or “potassium” and E2 is “channel” or “channels”, the M is regarded as a substring.
